# Uncertainties on $\alpha _S$ in the MMHT2014 global PDF analysis and implications for SM predictions

**DOI:** 10.1140/epjc/s10052-015-3630-3

**Published:** 2015-09-21

**Authors:** L. A. Harland-Lang, A. D. Martin, P. Motylinski, R. S. Thorne

**Affiliations:** Department of Physics and Astronomy, University College London, London, WC1E 6BT UK; Institute for Particle Physics Phenomenology, Durham University, Durham, DH1 3LE UK

## Abstract

We investigate the uncertainty in the strong coupling $\alpha _S(M_{Z}^{2})$ when allowing it to be a free parameter in the recent MMHT global analyses of deep-inelastic and related hard scattering data that was undertaken to determine the parton distribution functions (PDFs) of the proton. The analysis uses the standard framework of leading twist fixed-order collinear factorisation in the ${\overline{\mathrm{MS}}}$ scheme. We study the constraints on $\alpha _S(M_{Z}^{2})$ coming from individual data sets by repeating the NNLO and NLO fits spanning the range 0.108 to 0.128 in units of 0.001, making all PDFs sets available. The inclusion of the cross section for inclusive $t\bar{t}$ production allows us to explore the correlation between the mass $m_t$ of the top quark and $\alpha _S(M_{Z}^{2})$. We find that the best-fit values are $\alpha _S(M_{Z}^{2})=0.1201\pm 0.0015$ and $0.1172\pm 0.0013$ at NLO and NNLO, respectively, with the central values changing to $\alpha _S(M_Z^2)=0.1195$ and 0.1178 when the world average of $\alpha _S(M_{Z}^{2})$ is used as a data point. We investigate the interplay between the uncertainties on $\alpha _S(M_{Z}^{2})$ and on the PDFs. In particular we calculate the cross sections for key processes at the LHC and show how the uncertainties from the PDFs and from $\alpha _S(M_{Z}^{2})$ can be provided independently and be combined.

## Introduction

There has been a continual improvement in the precision and in the variety of the data for deep-inelastic and related hard-scattering processes. Noteworthy additions in the years since the MSTW2008 analysis [1] have been the HERA combined H1 and ZEUS data on the total [2] and charm structure functions [3], and the variety of new data sets obtained at the LHC, as well as updated Tevatron data (for full references see [4]). Moreover, the procedures used in the global PDF analyses of these data have been refined, allowing the partonic structure of the proton to be determined with ever-increasing accuracy and reliability. These improvements are important as it is necessary to quantify the Standard Model background as accurately as possible in order to isolate possible experimental signals of New Physics. One area that needs careful attention, at the present level of accuracy, is the treatment of the strong coupling, $\alpha _S$ itself, in the global analyses. Here we extend the recent MMHT2014 global PDF analysis [4] to study the uncertainties on $\alpha _S$ and their implications for predictions for processes at the LHC.

## Treatment of $\alpha _S(M_Z^2)$ in the MMHT2014 analysis

**Fig. 1:**
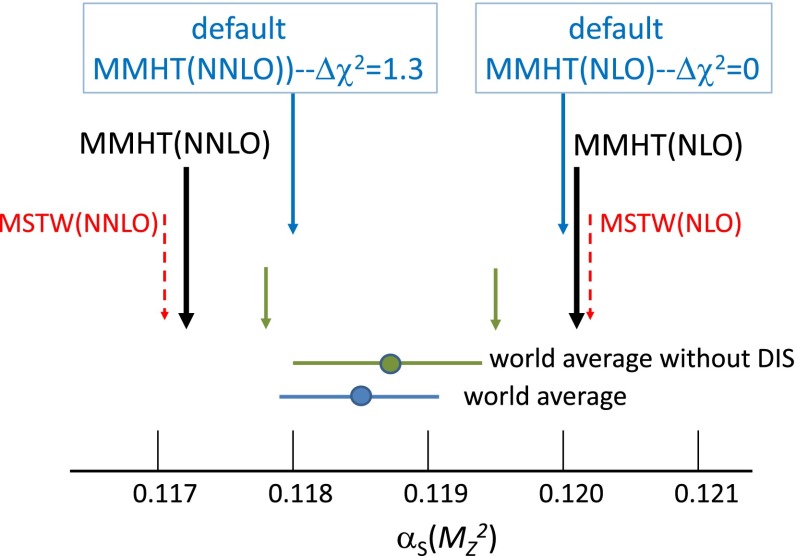
The *dark arrows* indicate the optimal values of $\alpha _S(M_Z^2)$ found in NLO and NNLO fits of the MMHT2014 analysis [4]. The *dashed arrows* indicate the values found in the MSTW2008 analysis [1]. We also show the world average value, which we note was obtained assuming, for simplicity, that the NLO and NNLO values are the same – which, in principle, is not the case. The *short arrows* are also of interest as they indicate the NLO and NNLO values which would have been obtained from the MMHT2014 global analyses if the world average value (obtained without including DIS data) were to be included in the fit. However, the default values $\alpha _{S,\mathrm{NLO}}=0.120$ and $\alpha _{S,\mathrm{NNLO}}=0.118$ were selected for the final MMHT2014 PDF sets ‘for ease of use’; indeed, the small values of $\Delta \chi ^2$ are the minute changes in $\chi ^2_\mathrm{global}$ in going from the optimal to these default fits

**Fig. 2:**
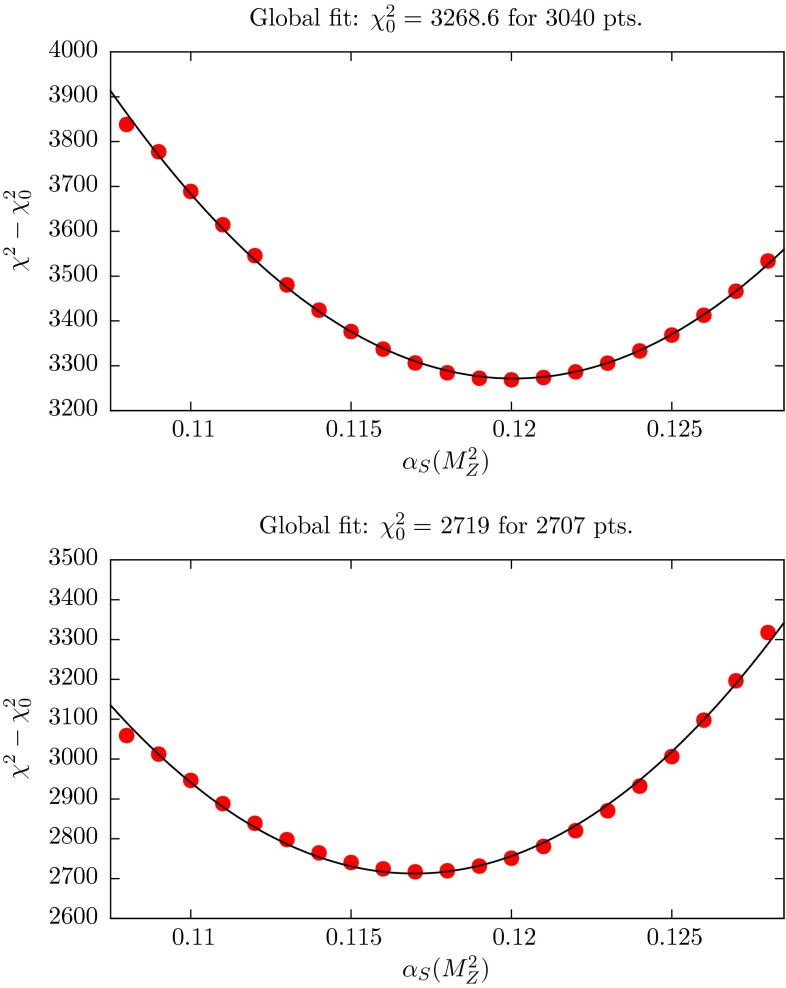
The *upper* and *lower plots* show total $\chi ^2$ as a function of the value of the parameter $\alpha _S(M_Z^2)$ for the NLO and NNLO MMHT2014 fits, respectively

We refer to Fig. 1 for an overview of the treatment and of the values of $\alpha _S$ obtained in the MMHT2014 global PDF analysis [4]. At both NLO and NNLO the value of $\alpha _S(M_Z^2)$ is allowed to vary just as another free parameter in the global fit. The best-fit values are found to be

1 $$\begin{aligned} \alpha _{S,\mathrm{NLO}}(M_Z^2)= & {} 0.1201 \end{aligned}$$

2 $$\begin{aligned} \alpha _{S,\mathrm{NNLO}}(M_Z^2)= & {} 0.1172, \end{aligned}$$

as indicated by the dark arrows in Fig. 1. The corresponding total $\chi ^2$ profiles versus $\alpha _S(M_Z^2)$ are shown in Fig. 2. These plots clearly show the reduction in the optimum value of $\alpha _S(M_Z^2)$ as we go from the NLO to the NNLO analysis. In the next section we show how the individual data sets contribute to make up this $\chi ^2$ profile versus $\alpha _S(M_Z^2)$.

It is sometimes debated whether one should attempt to extract the value of $\alpha _S(M_Z^2)$ from the PDF global fits or to simply use a fixed value taken from elsewhere – for example, to use the world average value [5]. However, we believe that very useful information on the coupling can be obtained from PDF fits, and hence have performed fits where this is left as a free parameter. As the extracted values of $\alpha _S(M_Z^2)$ in the NLO and NNLO MMHT2014 analyses [4] reasonably bridge the world average of $\alpha _S(M_Z^2)=0.1185\pm 0.0006$ [5], we regard these as our best fits. We note it is a common result in PDF analyses, and in other extractions of the strong coupling, for the best-fit value to fall slightly as the order of the theoretical calculations increases. However, in order to explore further, as well as leaving $\alpha _S(M^2_Z)$ as a completely independent parameter, the MMHT2014 analyses were repeated including the world average value (without the inclusion of DIS data to avoid double counting) of $\alpha _S(M_Z^2)=0.1187\pm 0.0007$ as a data point in the fit. This changed the preferred values to

3 $$\begin{aligned} \alpha _{S,\mathrm{NLO}}(M^2_Z)=0.1195 \quad \mathrm{and} \quad \alpha _{S,\mathrm{NNLO}}(M^2_Z)=0.1178,\nonumber \\ \end{aligned}$$

as indicated by the short arrows in Fig. 1. Each of these is about one standard deviation away from the world average, so our PDF fit is entirely consistent with the independent determinations of the coupling. Moreover, the quality of the fit to the data (other than the single ‘data’ point on $\alpha _S(M_Z^2)$) increases by about 1.5 units in $\chi ^2$ at NLO and just over one unit at NNLO when the coupling was included as a data point.

However, ultimately for the use of PDF sets by external users it is preferable to present the sets at common (and hence ‘rounded’) values of $\alpha _S(M_Z^2)$ in order to compare and combine with PDF sets from other groups, for example as in [6–9]. At NLO in the MMHT2014 analysis [4] we hence chose $\alpha _S(M_Z^2)=0.120$ as the default value for which the PDF sets with full error eigenvectors are made available. This is essentially identical to the value for the best PDF fit when the coupling is free, and still very similar when the world average was included as a constraint. At NNLO, $\alpha _S(M_Z^2)=0.118$ was chosen as a rounded value, very close to both the best-fit value and the world average, and the fit quality is still only 1.3 units in $\chi ^2$ higher than that when the coupling was free. This is extremely close to the value determined when the world average is included as a data point. Hence, in MMHT2014 [4], we chose to use $\alpha _S(M_Z^2)=0.118$ as the default for NNLO PDFs, a value which is very consistent with the world average. At NLO we also made a set available with $\alpha _S(M_Z^2)=0.118$, but in this case the $\chi ^2$ increases by 17.5 units from the best-fit value. In [4] we also made available PDF sets corresponding to the best fit for $\alpha _S(M_Z^2)$ values $\pm 0.001$ relative to the default values in order for users to determine the $\alpha _S(M_Z^2)$-uncertainty in predictions if so desired. We will return to the issue of PDF+$\alpha _S(M_Z^2)$ uncertainty later.

Before we continue we should specify how the running of $\alpha _S(Q^2)$ is treated. There is more than one definition of the coupling commonly used in QCD phenomenology. Although the various prescriptions are all formally equivalent since they differ only at higher orders, numerical differences of the order of up to 1 $\%$ can occur. We use the definition based on the full solution of the renormalisation group equation, in $\overline{\mathrm{MS}}$ scheme, at the appropriate order, with boundary condition defined by the value of $\alpha _S(M_Z^2)$. This is identical to the definition in public codes such as pegasus [10] and hoppet [11], and it is now effectively the standard in PDF analyses.^1^ It differs, for example, from solutions to the renormalisation group equations truncated at a particular order.

## Description of data sets as a function of $\alpha _S$

The NNLO MMHT2014 global analysis [4] was based on a fit to 40 different sets of data on deep-inelastic and related hard scattering processes. There were 10 different data sets of structure functions from the fixed-target charged lepton–nucleon experiments of the SLAC, BCDMS, NMC and E665 collaborations, six different neutrino data sets on $F_2,~xF_3$ and dimuon production from the NuTeV, CHORUS and CCFR collaborations, two Drell–Yan data sets from E886/NuSea, six different data sets from HERA involving the combined H1 and ZEUS structure function data, seven data sets from the Tevatron giving the measurements of inclusive jet, *W* and *Z* production by the CDF and D0 collaborations and, finally, nine data sets from the ATLAS, CMS and LHCb collaborations at the LHC. In addition, the NLO fit also used jet data from the ATLAS, CMS and H1 and ZEUS collaborations, which were not used at NNLO because it was judged that at present there is not sufficient knowledge of the full jet NNLO cross section; jet production at the Tevatron, on the other hand, is much closer to threshold than at the LHC, so the threshold approximation to the full NNLO calculation is much more likely to provide a reliable estimate in this case. The goodness-of-fit quantity, $\chi ^2_n$, for each of the data sets, $n=1,\ldots ,40$, is given for the NLO and NNLO global fits in Table 5 of [4], and the $\chi ^2$ definition is explained in Section 2.5 of the same article. The references to all the data that are fitted are also given in Ref. [4].

**Fig. 3:**
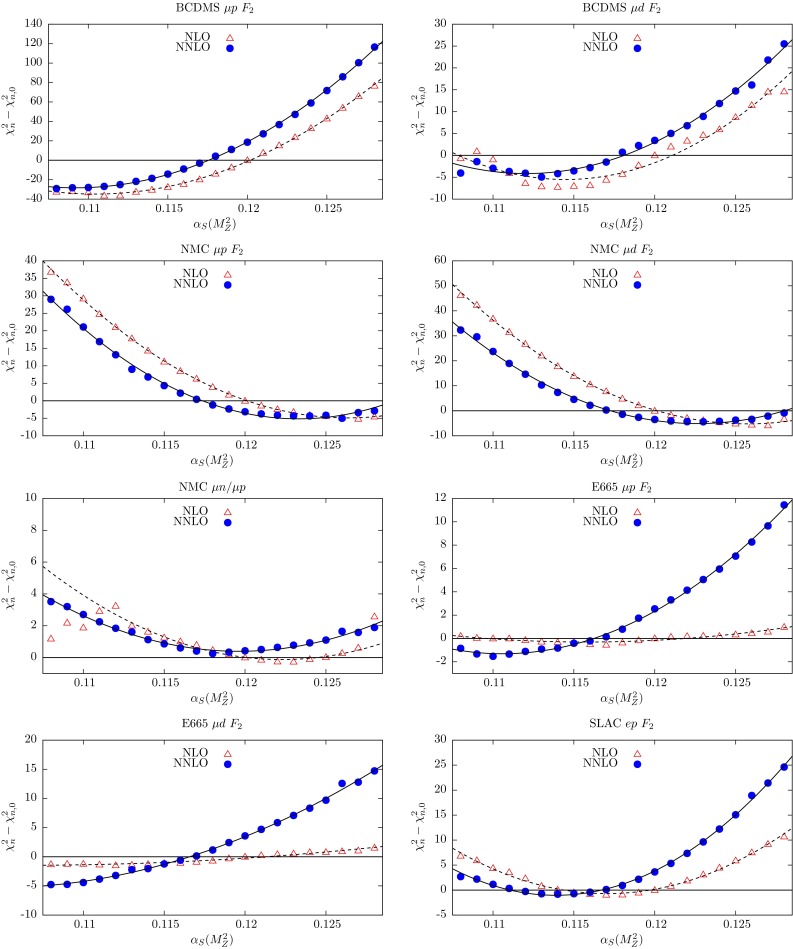
$\chi _n^2$ profiles obtained when varying $\alpha _S(M_Z^2)$ for the subset of data from deep-inelastic fixed-target experiments. The results from the NNLO global fits are shown by *bullet* points (and a *continuous curve*), while those from the NLO global fits are shown by *triangles* (and a *dashed curve*). The plots are continued in the next figure

**Fig. 4:**
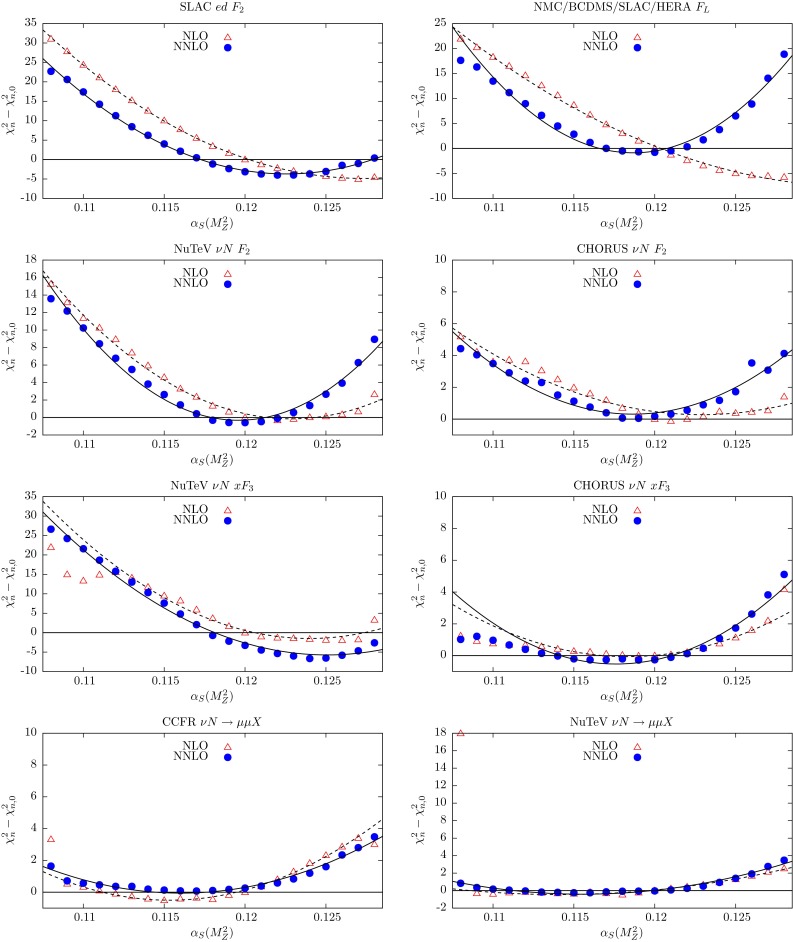
$\chi _n^2$ profiles obtained when varying $\alpha _S(M_Z^2)$, for the subset of data from deep-inelastic fixed-target experiments. The results from the NNLO global fits are shown by *bullet* points (and a *continuous curve*), while those from the NLO global fits are shown by *triangles* (and a *dashed curve*). (Continued from the previous figure)

**Fig. 5:**
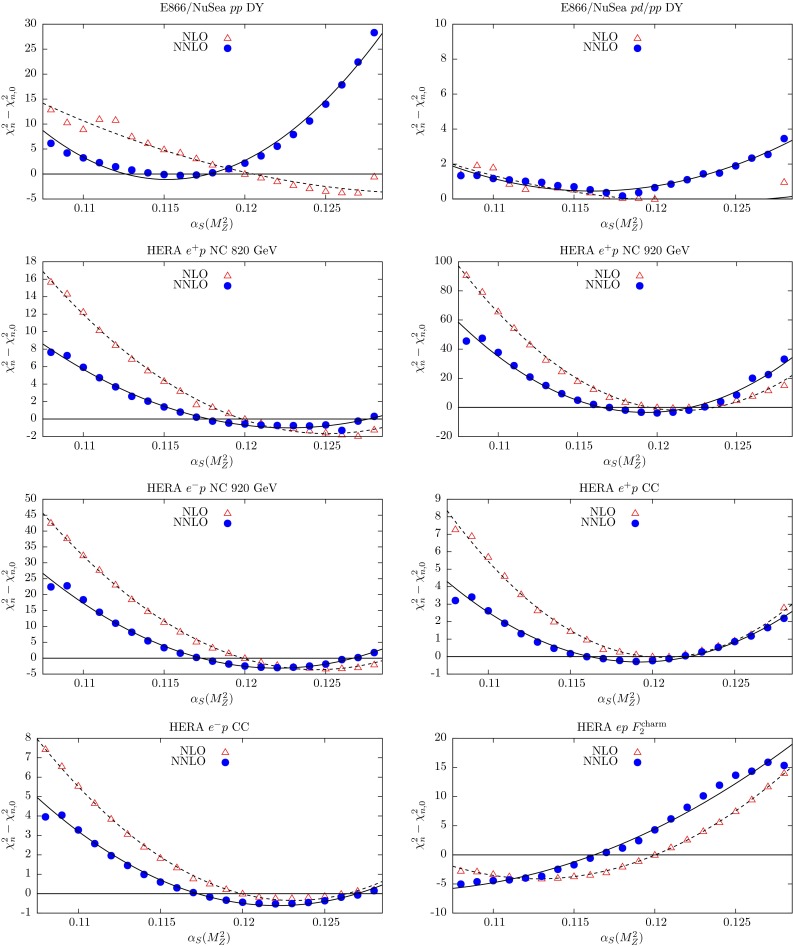
$\chi _n^2$ profiles obtained when varying $\alpha _S(M_Z^2)$ coming from the Drell–Yan fixed-target experiments and from the combined H1 and ZEUS measurements at HERA. The results from the NNLO global fits are shown by *bullet* points (and a *continuous curve*), while those from the NLO global fits are shown by *triangles* (and a *dashed curve*)

**Fig. 6:**
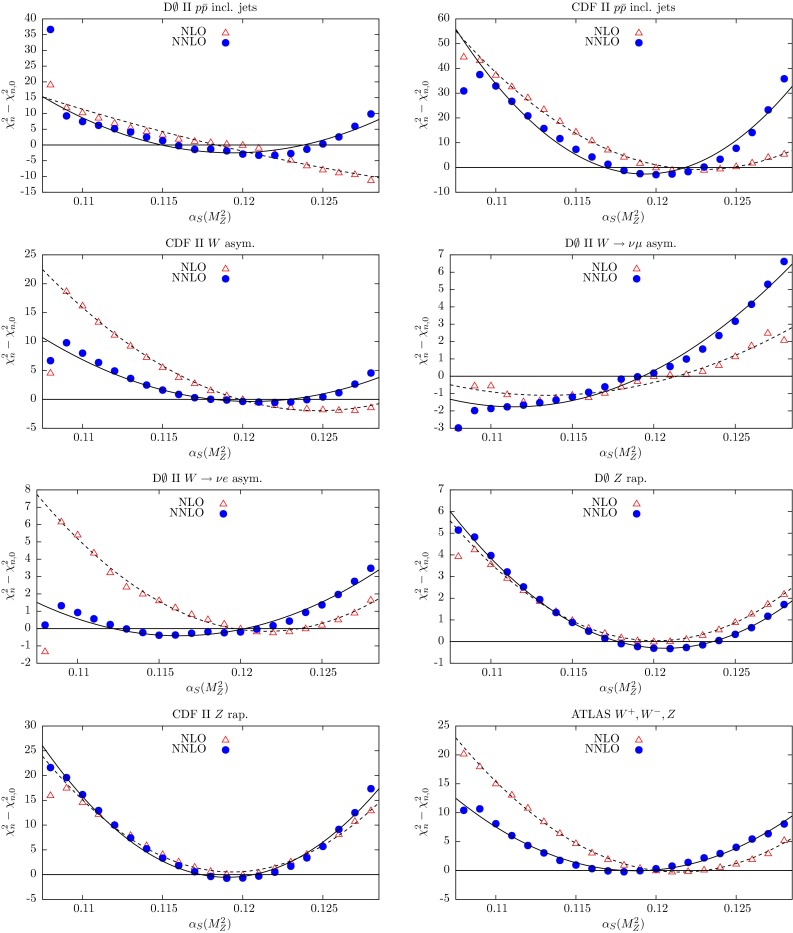
$\chi _n^2$ profiles obtained when varying $\alpha _S(M_Z^2)$, coming from the subset of data of the CDF and D0 Tevatron experiments, together with the plot for the ATLAS *W* and *Z* production data. The results from the NNLO global fits are shown by *bullet* points (and a *continuous curve*), while those from the NLO global fits are shown by *triangles* (and a *dashed curve*)

**Fig. 7:**
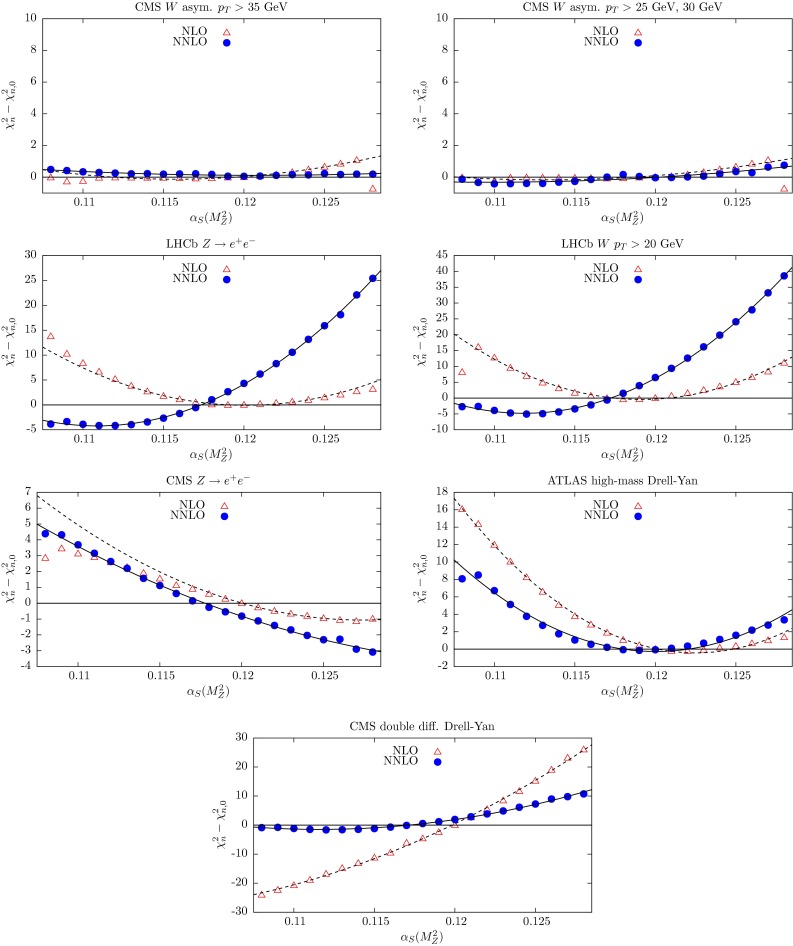
$\chi _n^2$ profiles obtained when varying $\alpha _S(M_Z^2)$, from the subset of data collected by the LHC experiments. The results from the NNLO global fits are shown by *bullet* points (and a *continuous curve*), while those from the NLO global fits are shown by *triangles* (and a *dashed curve*). The $\chi _n^2$ profiles for $t\overline{t}$ data are shown in Fig. 9 and discussed in Sect. 4

In the NNLO global fit of [4], let us denote the contribution to the total $\chi ^2$ from data set *n* by $\chi ^2_{n,0}$. Here we explore the $\chi ^2_n$ profiles as a function of $\alpha _S(M_Z^2)$ by repeating the global fit for different fixed values of $\alpha _S(M_Z^2)$ in the neighbourhood of the optimum value given in (2). The results are shown in Figs. 3, 4, 5, 6 and 7, where we plot the $\chi ^2_n$ profiles for each data set *n* as the difference from the value at the *global* minimum, $\chi ^2_{n,0}$, when varying $\alpha _S(M_Z^2)$. The points ($\bullet$) in Figs. 3, 4, 5, 6 and 7 are generated for fixed values of $\alpha _S(M_Z^2)$ between 0.108 and 0.128 in steps of 0.001. These points are then fitted to a quadratic function of $\alpha _S(M_Z^2)$ shown by the continuous curves. By definition we expect the profiles to satisfy $(\chi ^2_n-\chi ^2_{n,0})=0$ at $\alpha _S(M_Z^2)=0.1172$, corresponding to the value of $\alpha _S(M_Z^2)$ at the NNLO global minimum. Ideally, a data set should show a quadratic minimum about this point. Of course, in practice, the various data sets may pull, in varying degrees, to smaller or larger values of $\alpha _S(M_Z^2)$. There is a small amount of point-to-point fluctuation for the values of $(\chi ^2_n-\chi ^2_{n,0})$, even near the minimum, but near the minimum this is generally only at the level of fractions of a unit in $\chi ^2$ for a given data set. The fluctuations become larger as we go to values of $\alpha _S(M_Z^2)$ far from the minimum, particularly for lower $\alpha _S(M_Z^2)$, mainly because changes in $\chi ^2$ with small changes in $\alpha _S(M_Z^2)$ are becoming much greater. However, some of the “jumps” for individual sets near $\alpha _S(M_Z^2)=0.108$ imply that the global minimum in $\chi ^2$ for this choice of $\alpha _S(M_Z^2)$ is rather flat in certain parameter directions, with some relatively easy trade-off between the data sets which are poorly fit, and a transition to a different, approximately degenerate global minimum occurring with a small change in $\alpha _S(M_Z^2)$. Indeed, we have verified that at $\alpha _S(M_Z^2)=0.108$ there is a local minimum where “jumps” are eliminated, but with slightly higher global $\chi ^2$ than the result where there are “jumps”. This highlights the fact that the PDF uncertainty is difficult to define properly for a value of $\alpha _S(M_Z^2)$ which is far from optimal and leads to many data sets being badly fit.

**Fig. 8:**
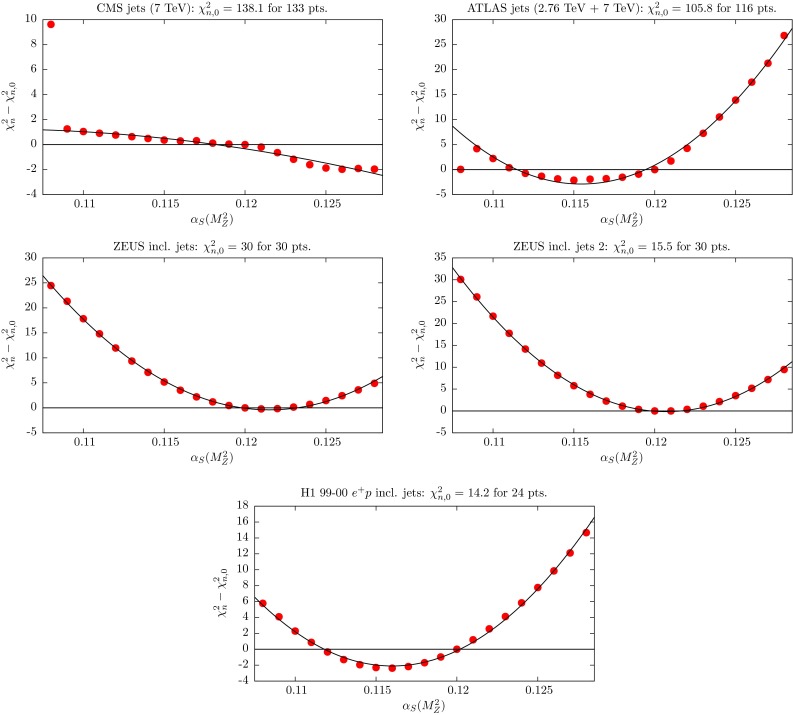
$\chi _n^2$ profiles for jet data sets, included in the NLO fit, but not in the NNLO fit, when varying $\alpha _S(M_Z^2)$

We repeat this exercise at NLO. Then the profiles will satisfy $(\chi ^2_n-\chi ^2_{n,0})=0$ at $\alpha _S(M_Z^2)=0.1201$. We include in the plots the NLO points (as triangles) and show the corresponding quadratic fit by a dashed curve. In Fig. 8 we show the $\chi ^2_n$ profiles for the LHC and HERA jet data that were included in the NLO fit. Here the bullet points and profile curve correspond to the NLO fit. These data were not included in the NNLO fit.

The fixed-target structure function data in the first 14 plots in Figs. 3 and 4 have been available for several years. These data play an important role in constraining the value of $\alpha _S(M_Z^2)$. There is some tension between these data sets. The BCDMS (and also the E665) data prefer values of $\alpha _S(M_Z^2)$ around 0.110. On the other hand the NMC data prefer values around 0.122; and the SLAC $F_2^{p,d}$ data prefer $\alpha _S(M_Z^2)$ values around 0.115 and 0.122, respectively. The neutrino $F_2$ and $xF_3$ data prefer $\alpha _S(M_Z^2) \sim 0.120$; while neutrino dimuon production has little dependence on $\alpha _S(M_Z^2)$, since the extra $B(D\rightarrow \mu )$ branching ratio parameter (see Eq. (19) of [4]) can partially compensate for the changes in $\alpha _S(M_Z^2)$.

The NNLO corrections to the structure functions are positive and speed up the evolution, leading to smaller optimum values of $\alpha _S(M_Z^2)$ than those at NLO, such that the spread of optimum values of $\alpha _S(M_Z^2)$ for the different data sets is somewhat reduced. Thus the overall fit to this subset of the data is marginally better at NNLO. The difference $\alpha _{S,\mathrm{NNLO}}<\alpha _{S,\mathrm{NLO}}$ is clearly evident in the majority of the corresponding plots.

The recent combined H1 and ZEUS structure function data from HERA prefer a value of $\alpha _S(M_Z^2)$ of about 0.120 at NNLO. Perhaps the only surprising result is the $\alpha _S(M_Z^2)$ behaviour of the combined data for $F_2^\mathrm{charm}$, which prefers a very low value of $\alpha _S(M_Z^2)$ at NNLO, whereas the uncombined data had a perfect quadratic behaviour about 0.118; see Fig. 5 of [13]. Note, however, that the combined data contains some points at the lowest $Q^2$ which were not available as an individual data set. These data, particularly at low $Q^2$, are sensitive to the value of the charm mass $m_\mathrm{c}$, and there is a correlation between its value and $\alpha _s(M_Z^2)$  [14]. This will be studied again with the up-to-date data in a future article.

The longitudinal structure function $F_L$ leads off with an $\alpha _S$ term, and so the value of $(\chi ^2_n-\chi ^2_{n,0})$ depends more sensitively on $\alpha _S(M_Z^2)$. The NNLO plot shows an excellent quadratic dependence on $\alpha _S(M_Z^2)$, centred at 0.118. The NNLO coefficient functions for $F_L(x,Q^2)$ [15, 16] are positive and significant, and the NLO fit tries to mimic these with a higher value of $\alpha _S(M_Z^2)$. Indeed, the data for $F_L$, and also the E866/NuSea *pp* Drell–Yan cross sections data, are clearly more quadratic at NNLO than at NLO, with minima closer to the best-fit values. This indicates a strong preference for the NNLO description, which is not so apparent if only the global best-fit values $\chi _{n,0}^2$ are known. As the E866/NuSea data for *pd* / *pp* Drell–Yan production are a ratio of cross sections, the sensitivity to the value of $\alpha _S(M_Z^2)$ is small.

The Tevatron data, as well as the ATLAS $W^{\pm },Z$ production data and the ATLAS high-mass Drell–Yan data, show, at NNLO, $\alpha _S(M_Z^2)$ profiles with quadratic behaviour with minima close to the best-fit values. Again, the profiles are improved to those at NLO. The counter example are the LHCb data, which have profiles which are more reasonable at NLO than at NNLO. In general, the charge-lepton asymmetry measurements arising from $W^{\pm }$ production at the Tevatron and the LHC, which are a ratio of cross sections, have much less constraint on the value of $\alpha _S(M_Z^2)$.

Judging from the values of $(\chi ^2_n-\chi ^2_{n,0})$ away from the different minima of the various data sets or, rather, the steepness of the quadratic forms in $\alpha _S(M_Z^2)$, we see that there is a tendency for data at lower energies or lower $Q^2$ to lead to more constraint on the optimum global value of $\alpha _S(M_Z^2)$. This is to be anticipated, as we will see in Sect. 6.

## $t\overline{t}$ data: $m_t$–$\alpha _S$ correlation

**Fig. 9:**
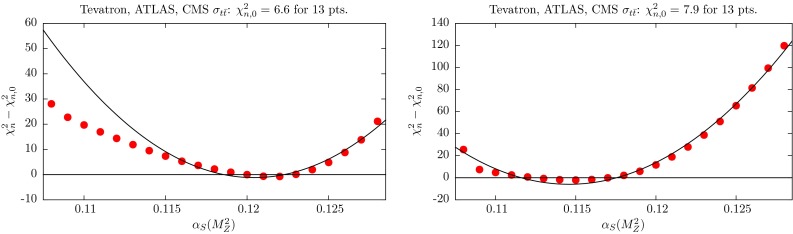
$\chi _n^2$ profiles for $t\overline{t}$ data in the NLO (*left*) and NNLO (*right*) fits, when varying $\alpha _S(M_Z^2)$

There is a particularly strong, but also complicated, relationship between the value of $\alpha _S(M_Z^2)$ and the fit to data on the inclusive cross section for ${t \bar{t}}$ production, $\sigma _{t \bar{t}}$. We show the $\chi ^2$ profiles at NLO and NNLO in Fig. 9. Clearly there is a preference for a lower value of $\alpha _S(M_Z^2)$ at NNLO than at NLO, and a strong constraint in both cases, with $\chi ^2$ increasing by a large number of units, certainly compared to the number of data points, for small changes in $\alpha _S(M_Z^2)$. Indeed, nominally $\sigma _{t\bar{t}}$ provides one of the strongest constraints of any data set for the lower limit of $\alpha _S(M_Z^2)$ at NLO and the upper limit of $\alpha _S(M_Z^2)$ at NNLO. However, the picture is more complicated than for other data sets due to the very strong correlation with the value of the mass $m_t$ of the top quark.

In the global fits the theory calculation of $\sigma _{t \bar{t}}$ is performed with a preferred value of the top-quark pole mass of $172.5~\mathrm GeV$, since this is the default in PYTHIA, used to extract the cross section in many of the measurements. Moreover, the majority of the cross sections are quoted for this value of $m_t$. This value is also consistent with the world average of the measured value of 173.34 GeV [5]. However, we allow a $1~\mathrm GeV$ uncertainty on the value of $m_t$, which can be thought of as accounting for the uncertainty in the value of $m_t$ itself and also for the small variation in the extracted cross sections with $m_t$ used; in general this is about a third the size of the variation of the calculation of $\sigma _{t \bar{t}}$ with $m_t$, and the net effect is an effective uncertainty a little lower than $1~\mathrm GeV$. To be specific, $m_t$ is left as a free parameter in the fit, but there is a $\chi ^2$ penalty of $\chi ^2_{m_t} = (m_t-172.5~\mathrm GeV)^2$ applied to keep the value close to the preferred value. This penalty is included in the values in Fig. 9. The allowed variation in $m_t$ away from the preferred central value of $172.5~\mathrm GeV$ results in the NLO fit preferring a low value of $m_t=171.7~\mathrm GeV$ and the NNLO fit preferring a high value of $m_t=174.2~\mathrm GeV$. The low value of $m_t$ in the global fit and the high value of $\alpha _S(M_Z^2)$ preferred by $\sigma _{t \bar{t}}$ when $\alpha _S(M_Z^2)$ is varied, both occur for the same reason. That is, the NLO cross section tends to undershoot the data, and raising $\alpha _S(M_Z^2)$ and lowering $m_t$ both raise the cross section, leading to better agreement.

The NNLO correction to the cross section in the pole mass scheme is moderate, but large compared to the most precise data, and hence the NNLO cross section tends to be too high. This leads to the opposite pulls to those at NLO, i.e., NNLO prefers $\alpha _S(M_Z^2)$ low and $m_t$ high. Within the global fit we find that the allowed variation with accompanying penalty for deviations from $m_t=172.5~\mathrm GeV$ results in $m_t$ values at the best-fit values of $\alpha _S(M_Z^2)$ which are of order 1–2$\sigma$ away from either our preferred value or the world average, so have no particular inconsistency, but it is useful to examine the interplay between $\alpha _S(M_Z^2)$ and $m_t$ in rather more detail.

### Effect on $\chi ^2_\mathrm{global}$ to changes of $m_t$ and $\alpha _S(M_Z^2)$

**Fig. 10:**
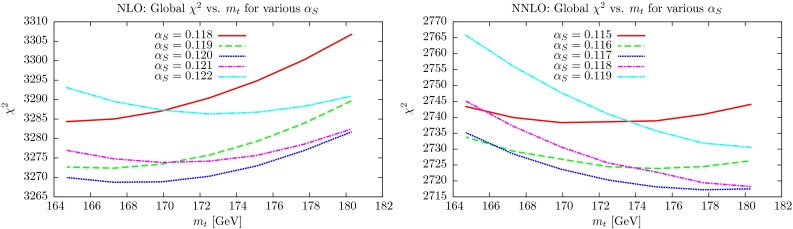
Global $\chi _n^2$ minima as a function of the top mass $m_t$, for different fixed values of $\alpha _S(M_Z^2)$. There is no $\chi ^2$ penalty for varying $m_t$

First we investigate the quality of the global fit as a function of both $\alpha _S(M_Z^2)$ and $m_t$. This is shown in Fig. 10, where we plot $\chi ^2_\mathrm{global}$ versus $m_t$ at several different values of $\alpha _S(M_Z^2)$ (In these plots $m_t$ is varied with no $\chi ^2$ penalty for deviations away from the “preferred” value). At NLO one can see that regardless of $m_t$ the best global fit is always obtained quite clearly for $\alpha _S(M_Z^2)$ close to 0.120, with the fit quality for $\alpha _S(M_Z^2)=0.119$ or $\alpha _S(M_Z^2)=0.121$ each being a few units worse at all values of $m_t$. It is only for $m_t > 180~\mathrm GeV$ that the quality for $\alpha _S(M_Z^2)=0.121$ approaches that of 0.120 and the best fit would be for $\alpha _S(M_Z^2)\approx 0.1205$. At this mass the global $\chi ^2$ is about 10 units above the minimum though. Similarly at NNLO $\alpha _S(M_Z^2)=0.117$ gives a lower $\chi ^2_\mathrm{global}$ for all masses between about $166~\mathrm GeV$ and $181~\mathrm GeV$, when $\alpha _S(M_Z^2)=0.116$ and $\alpha _S(M_Z^2)=0.118$, respectively, give the same $\chi ^2_\mathrm{global}$ values. Hence, even completely unreasonable variations of $\sim$7–10 GeV result in changes of the best-fit values of $\alpha _S(M_Z^2)$ of only $\sim$0.0005. We do note, however, that without a penalty for $m_t$ variation the best global fits are at $m_t=168~\mathrm GeV$ and $m_t=180~\mathrm GeV$ at NLO and NNLO, respectively, so some penalty is clearly necessary. Ultimately, the value of $\alpha _S(M_Z^2)$ determined by the global fit is very insensitive to the value of $m_t$ used and, indeed, to the $\sigma _{t \bar{t}}$ data, because these correspond to relatively few data points. Indeed, if these are left out of the global fit the change in the optimum value of $\alpha _S(M_Z^2)$ is only of order $0.0001-2$ at NLO and NNLO. However, the interplay between $\alpha _S(M_Z^2)$ and $m_t$ is more dramatic for the $\sigma _{t \bar{t}}$ data alone, as we will now show.

### Effect on $\chi ^2_{t \bar{t}}$ to changes of $m_t$ and $\alpha _S(M_Z^2)$

**Fig. 11:**
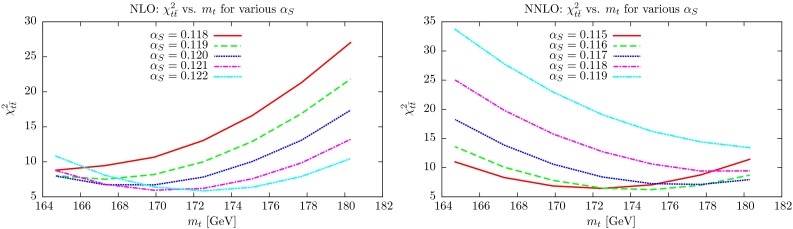
$\chi _n^2$ values for inclusive $t\overline{t}$ cross section data at the global minimum, as a function of the top mass $m_t$, for different fixed values of $\alpha _S(M_Z^2)$. There is no $\chi ^2$ penalty for varying $m_t$

The equivalent plots to Fig. 10 are shown in Fig. 11 for the fit quality to the inclusive $\sigma _{t \bar{t}}$ cross section data. Again, there is no penalty applied for $m_t$ variation. At NLO it is clear that, except for very low values of $m_t$, the best fit is achieved for higher values of $\alpha _S(M_Z^2)$, i.e. $\alpha _S(M_Z^2)=0.121$ or for $m_t>172~\mathrm GeV$, $\alpha _S(M_Z^2)=0.122$. Indeed, the best possible fit to the top cross section data is for $m_t\approx 172~\mathrm GeV$ and $\alpha _S(M_Z^2)=0.122$. However, the improvement in $\chi ^2_{t\bar{t}}$ compared to $\alpha _S(M_Z^2)=0.120$ for this mass is only $\sim$2 units – far less than the deterioration in the $\chi ^2$ for the rest of the data when going from $\alpha _S(M_Z^2)=0.120$ to 0.122. Overall the minimum $\chi ^2$ achieved for any $\alpha _S(M_Z^2)$ is quite flat with $m_t$, changing by at most 2 units for $168~\mathrm GeV< { m}_{ t} < 178~\mathrm GeV$. However, it is clear that the variation of $\chi ^2_{t \bar{t}}$ is different for different values of $\alpha _S(M_Z^2)$. As $\alpha _S(M_Z^2)$ decreases there is a preference for a smaller mass, hence if the central value of $m_t$ had been chosen higher than $172.5~\mathrm GeV$ for example, the best fit to $\sigma _{t \bar{t}}$ would be for a higher value of $\alpha _S(M_Z^2)$. The constraint on $\alpha _S(M_Z^2)$ in the upper direction would be weakened slightly; however, this data set does provide a significant constraint in this direction. If the penalty had been less severe, e.g. an increase in $\chi ^2_{t \bar{t}}$ for $\Delta m_t =2~\mathrm GeV$ rather than $\Delta m_t=1~\mathrm GeV$, the best value of $m_t$ and $\alpha _S(M_Z^2)$ would not change significantly, as the fit quality does not improve for masses of $m_t<171.7$ for any $\alpha _S(M_Z^2)$, even discounting the penalty. However, the $\chi ^2_{t \bar{t}}$ curves for lower values of $\alpha _S(M_Z^2)$, i.e. 0.119 and 0.118 are falling quite steeply as $m_t$ decreases in the vicinity of $m_t=172~\mathrm GeV$, so the increase in $\chi ^2_{t\bar{t}}$ with decreasing $\alpha _S(M_Z^2)$ seen in Fig. 9 (left) would be less severe if for $m_t$ it was allowed to choose smaller values, and the constraint on the lower values of $\alpha _S(M_Z^2)$ would be reduced somewhat. Hence, at NLO, alternative treatments of $m_t$ would allow a slightly higher best fit $\alpha _S(M_Z^2)$ than the default treatment, and a little scope for a relaxation of the lower limit on $\alpha _S(M_Z^2)$.

At NNLO it is again clear that higher values of $$\alpha _S(M_Z^2)$$ prefer higher values of $$m_t$$. However, for $$\alpha _S(M_Z^2)=0.118$$ or 0.119 the value of $$m_t$$ corresponding to the best fit is $$m_t=180~\mathrm GeV$$ or more. Again, there is little variation in the best value of $$\chi ^2_{t\bar{t}}$$ for $$168~\mathrm GeV$$$$ < m_t < 178~\mathrm GeV$$, but the best fit is achieved for $$\alpha _S(M_Z^2)=0.115$$ or 0.116,^2^ only becoming $$\alpha _S(M_Z^2)=0.117$$ at $$m_t=178~\mathrm GeV$$. In this case if the penalty for variations in $$m_t$$ away from the default central value were relaxed it would make little difference, as even for $$\alpha _S(M_Z^2)=0.115$$ the best fit is for $$m_t\approx 172 ~\mathrm GeV$$. It might allow slightly better fits for $$\alpha _S(M_Z^2)\sim 0.110$$, but this would have no influence on the overall constraint on $$\alpha _S(M_Z^2)$$, which is constrained by many data not to be much lower than 0.115. A potential increase in $$m_t$$, either by change of default central value, or a relaxation of the penalty, would allow for a potentially a slightly higher value of $$m_t$$ for the best fit, as the minimum possible $$\chi ^2_{t\bar{t}}$$ is almost completely flat between $$172~\mathrm GeV$$$$<m_t < 176~\mathrm GeV$$. This would be accompanied by a slight increase in $$\alpha _S(M_Z^2)$$. It would also allow a little relaxation in the constraint on higher values of $$\alpha _S(M_Z^2)$$. The $$\chi ^2_{t \bar{t}}$$ curves for $$\alpha _S(M_Z^2)=0.118$$ and 0.119 are decreasing with increasing $$m_t$$ in the vicinity of $$m_t=174~\mathrm GeV$$, and a higher allowed value of $$m_t$$ would enable the increase in $$\chi ^2$$ with $$\alpha _S(M_Z^2)$$ in Fig. 11 (right) to be less steep. Hence, at NNLO alternative treatments of $$m_t$$ would allow a slightly higher best-fit value of $$\alpha _S(M_Z^2)$$ than the default treatment, and a little scope for a relaxation of the upper limit on $$\alpha _S(M_Z^2)$$.

Hence, the overall conclusion is that some added freedom in $m_t$ would lead to potentially rather small changes in the minima of the $\chi ^2$ curves in Fig. 11, but a reduced rate of increase of $\chi ^2$ away from the minima. The implications of this will be discussed in the next section.

## Uncertainty on $\alpha _S(M_Z^2)$ and calculation of PDF+$\alpha _S(M_Z^2)$ uncertainty

First, recall that in the MMHT2014 analysis [4] we determined the uncertainties of the PDFs using the Hessian approach with a dynamical tolerance procedure. We obtained PDF ‘error’ eigenvector sets, each corresponding to 68 $\%$ confidence level uncertainty, where the vectors are orthogonal to each other and span the PDF parameter space.

**Fig. 12:**
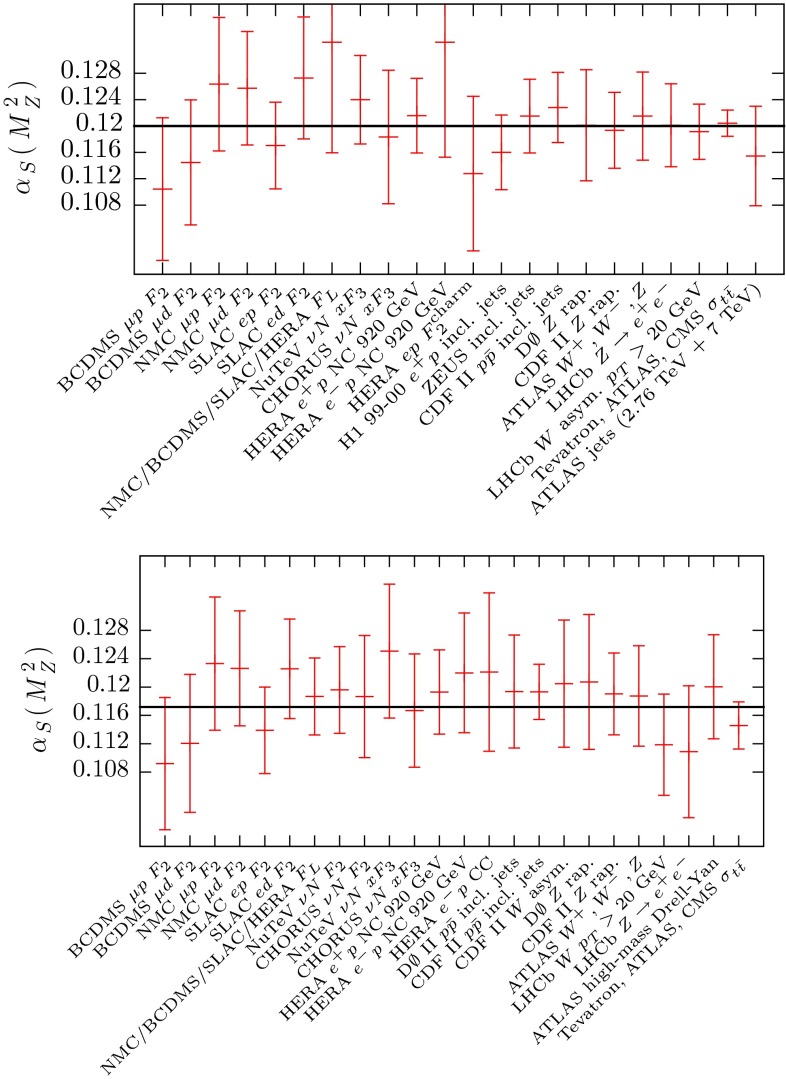
The *upper* and *lower* plots show the value of $\alpha _S(M_Z^2)$ corresponding to the best fit, together with the upper and lower 1$\sigma$ constraints on $\alpha _S(M_Z^2)$ from the more constraining data sets at NLO and NNLO, respectively

In order to determine the uncertainty on $\alpha _S(M_Z^2)$ at NLO and NNLO we begin by using the same technique as in the MSTW study of Ref. [13]; that is, for the ‘error’ eigenvectors we apply the tolerance procedure to determine the uncertainty in each direction away from the value at the best fit when one data set goes beyond its 68 % confidence level uncertainty. The values at which each data set does reach its 68 % confidence level uncertainty, plus the value of $\alpha _S(M_Z^2)$ for which each data set has its best fit (within the context of a global fit) are shown at NLO and NNLO in Fig. 12. However, unlike Fig. 7 of [13] we do not show all data, as with the increased number of sets there are now too many to show clearly on a single figure. Moreover, as seen earlier, many data sets have very little dependence, and hence produce very little constraint. Hence, we show those where both limits are within the range of $\alpha _S(M_Z^2)$ explicitly studied, i.e. $0.108-0.128$ or where one limit is within 0.005 of the best-fit value of $\alpha _S(M_Z^2)$. None of the data sets omitted using these criteria have a significant pull on $\alpha _S(M_Z^2)$.

The dominant constraint on $\alpha _S(M_Z^2)$ in the downwards direction at NLO is from the top pair cross section data and, using the dynamical tolerance procedure, gives an uncertainty of $\Delta \alpha _S(M_Z^2)=-0.0014$. In the upwards direction it is the BCDMSp data with an uncertainty of $\Delta \alpha _S(M_Z^2)= +0.0012$. At NNLO the dominant downward constraint comes from NuTeV $F_3(x,Q^2)$ data which gives $\Delta \alpha _S(M_Z^2)= - 0.0012$ and in the upwards direction it is the top pair cross section data, where the uncertainty is $\Delta \alpha _S(M_Z^2)=+0.0008$.

There are a number of other data sets which give almost as strong constraints. For instance, at NLO in the downwards direction we find that SLAC deuterium data give $\Delta \alpha _S(M_Z^2)= -0.0018$ and in the upwards direction H1 jets give $\Delta \alpha _S(M_Z^2)= +0.0019$. At NNLO in the downwards direction SLAC deuterium data and CDF jet data give $\Delta \alpha _S(M_Z^2)\approx -0.0014$, and in the upwards direction, at NNLO, the BCDMSp data give $\Delta \alpha _S(M_Z^2)=+0.0014$. In all cases there are other data sets that are not much less constraining than those mentioned explicitly. Hence, in no case is it a single data set which is overwhelmingly providing the dominant constraint on the upper or lower limit of $\alpha _S(M_Z^2)$. Similarly, no single data sets would change the central value by more than 0.001 if it were to be omitted.

Two of the four dominant constraints nominally come from $\sigma _{t \bar{t}}$, and at NLO we have $\alpha _S(M_Z^2)=0.1201_{-0.0014}^{+0.0012}$ and at NNLO $\alpha _S(M_Z^2)= 0.1172_{-0.0012}^{+0.0008}$. However, in the previous section we highlighted the interplay between $\alpha _S(M_Z^2)$ and $m_t$ when examining the fit quality of the $\sigma _{t \bar{t}}$ data. We demonstrated that if some extra flexibility is allowed on the choice of central value of $m_t$ and/or on the 1-$\sigma$ uncertainty that is used, then the constraints are relaxed to some degree. Hence, we are reluctant to treat the constraint from the data on $\sigma _{t \bar{t}}$ completely rigorously. In order to see quite how we should deal with the constraints nominally due to these data, we first check which data sets provide the next tightest constraint. If we were simply to ignore the constraints from $\sigma _{t \bar{t}}$ we would find a change in uncertainty at NLO of $\Delta \alpha _S(M_Z^2)= -0.0012 \rightarrow -0.0017$ and at NNLO $\Delta \alpha _S(M_Z^2)= +0.0008 \rightarrow +0.0014$. These are significant, but hardly dramatic changes, and it would be no surprise if some alternative treatment of the default top mass resulted in changes of a similar type. Hence, it might be suitable to take these values as a simple alternative, arguing that the constraints from $\sigma _{t \bar{t}}$ are not sufficiently greater than those from other data sets *either* to ignore the possible effects of alternative treatments of the mass $m_t$*or* to warrant a completely thorough investigation at this stage.^3^ However, there is the additional feature to note – whichever criterion we use, we have some, albeit not too dramatic, asymmetry in the $\alpha _S(M_Z^2)$ uncertainty. There is no strong reason to apply this slight asymmetry, as the $\chi ^2$ profile for the global fit follows the quadratic curve very well at both NLO and NNLO, and the degree of asymmetry obtained using the dynamical tolerance procedure is arguably within the “uncertainty of the uncertainty”. Hence at NLO and NNLO we average the two uncertainties (obtained without the $\sigma _{t \bar{t}}$ constraint) obtaining

4 $$\begin{aligned} \alpha _{S,\mathrm{NLO}}(M_Z^2)= & {} 0.1201 \pm 0.0015 \end{aligned}$$

5 $$\begin{aligned} \alpha _{S,\mathrm{NNLO}}(M_Z^2)= & {} 0.1172 \pm 0.0013. \end{aligned}$$

This corresponds to $\Delta ^\mathrm{NLO} \chi ^2_\mathrm{global}=10.3$ and $\Delta ^\mathrm{NNLO} \chi ^2_\mathrm{global}=7.2$. These are the sort of tolerance values typical of the majority of PDF eigenvectors.

Each of these values of $\alpha _S(M_Z^2)$ is within 1$\sigma$ of the world average (without DIS data) of $0.1187 \pm 0.0007$, though in opposite directions. As noted earlier, the inclusion of $\alpha _S(M_Z^2)$ as a data point leads to values of 0.1178 and 0.1195 at NNLO and NLO, respectively. These are somewhat closer to the world average, and very near to 0.118 at NNLO, but still quite close to 0.120 at NLO.^4^ Hence, we interpret the values in Eqs. (4) and (5) as independent measurements of $\alpha _S(M_Z^2)$, but acknowledge that at NNLO taking both this determination and the world average into account a round value of $\alpha _S(M_Z^2)=0.118$ is an appropriate one at which to present the PDFs. At NLO we would recommend the use of $\alpha _S(M_Z^2)=0.120$ as the preferred value for the PDFs, but have made eigenvector sets available at $\alpha _S(M_Z^2)=0.118$. If a value of $\alpha _S(M_Z^2)=0.119$ were desired the average of the results at $\alpha _S(M_Z^2)=0.118$ and 0.120 would provide an excellent approximation.

When considering the uncertainty on the prediction for a physical quantity we should include the uncertainty on $\alpha _S(M_Z^2)$, as well as that on the PDFs. This is particularly important for cross sections that at leading order are proportional to a power of the coupling, such as $\sigma _{t{\bar{t}}}$ or $\sigma _\mathrm{Higgs}$, which are proportional to $\alpha _S^2$. A naive procedure would be to compute the error as

6 $$\begin{aligned} \Delta \sigma =\sqrt{(\Delta \sigma _\mathrm{PDF})^2+(\Delta \sigma _{\alpha _S})^2} \end{aligned}$$

where $\Delta \sigma _{\alpha _S}$ is the variation of the cross section when $\alpha _S(M_Z^2)$ is allowed to vary over a given range. However, it is inconsistent to use different values of $\alpha _S$ in the partonic hard subprocess cross section and in the PDF evolution. Moreover, in a global PDF analysis, there are non-negligible correlations between the PDFs and the value of $\alpha _S$.

In the MSTW study [13] of the PDF$+\alpha _S(M_Z^2)$ uncertainties arising from the MSTW2008 analysis we advocated using our best fit value of $\alpha _S(M_Z^2)$ as the central value for PDF predictions, and then provided additional eigenvector sets at $\pm 0.5 \sigma$ and $\pm 1 \sigma$ values of $\alpha _S(M_Z^2)$. The uncertainty was then calculated by taking the envelope of the predictions using all these eigenvector sets. This still seems like an appropriate algorithm for use with the dynamical tolerance procedure of obtaining uncertainties. However, it can only really be applied if the central prediction is obtained using the PDFs defined at the best-fit value of $\alpha _S(M_Z^2)$, which is no longer the case, and, moreover, was a rather complicated and time-consuming procedure.

Since the MSTW study [13] was undertaken it has been shown that, within the Hessian approach to PDF uncertainties, the correct PDF+$\alpha _S(M_Z^2)$ uncertainty on any quantity can be obtained by simply taking the PDFs defined at $\alpha _S(M_Z^2)\pm \Delta \alpha _S(M_Z^2)$ and treating these two PDF sets (and their accompanying value of $\alpha _S(M_Z^2)$) as an extra pair of eigenvectors [18]. In short, the full uncertainty is obtained by adding the uncertainty from this extra eigenvector pair in quadrature with the PDF uncertainty. So we are back to the naive form (6), but now, importantly, with the correlations between the PDFs and $\alpha _S$ included. This has the advantages of both being very simple, but also separating out the $\alpha _S(M_Z^2)$ uncertainty on a quantity explicitly from the purely PDF uncertainty. Strictly speaking, the method only holds if the central PDFs are those obtained from the best fit when $\alpha _S(M_Z^2)$ is left free, and if the uncertainty $\Delta \alpha _S(M_Z^2)$ on $\alpha _S(M_Z^2)$ that is used is the uncertainty obtained from the fit. If we use PDFs defined at $\alpha _S(M_Z^2)=0.118$ at NNLO we are still very near the best fit, and the error induced will be very small. At NLO a larger error will be induced by using the PDFs defined at $\alpha _S(M_Z^2)=0.118$ than those at $\alpha _S(M_Z^2)=0.120$. Any choice of $\Delta \alpha _S(M_Z^2)$ of $0.001-0.002$ should only induce a small error. Hence, overall we now advocate using this approach with NLO PDFs defined at $\alpha _S(M_Z^2)=0.120$ and NNLO PDFs defined at $\alpha _S(M_Z^2)=0.118$. The value of $\Delta \alpha _S(M_Z^2)$ is open to the choice of the user to some extent, but it is recommended to stay within the range $\Delta \alpha _S(M_Z^2)$ that we have found.

In Sect. 7 we apply the above procedure to determination of the PDF$+\alpha _S(M_Z^2)$ uncertainties on the predictions for the cross sections for benchmark processes at the Tevatron and the LHC, but first we examine the change in the PDF sets themselves with $\alpha _S(M_Z^2)$.

## Comparison of PDF sets 

**Fig. 13:**
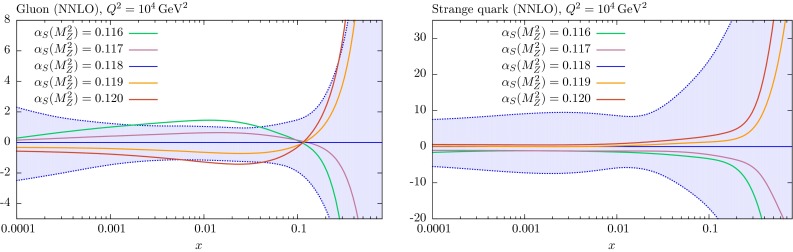
Percentage difference in the NNLO gluon and strange-quark PDFs at $Q^2=10^4$ $\mathrm{GeV}^2$ relative to central ($\alpha _S(M_Z^2)=0.118$) set for fits with different values of $\alpha _S$, with the percentage error bands for the central set also shown

**Fig. 14:**
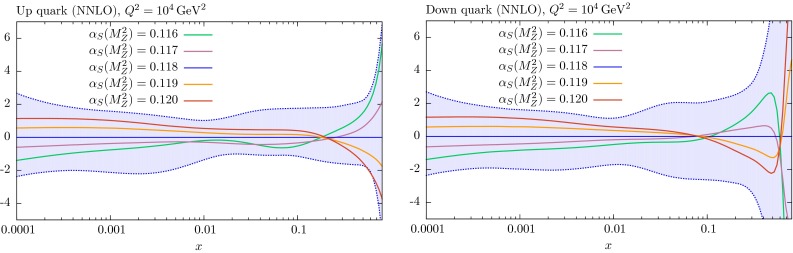
Percentage difference in the NNLO up and down quark PDFs at $Q^2=10^4$ $\mathrm{GeV}^2$ relative to central ($\alpha _S(M_Z^2)=0.118$) set for fits with different values of $\alpha _S$, with the percentage error bands for the central set also shown

**Fig. 15:**
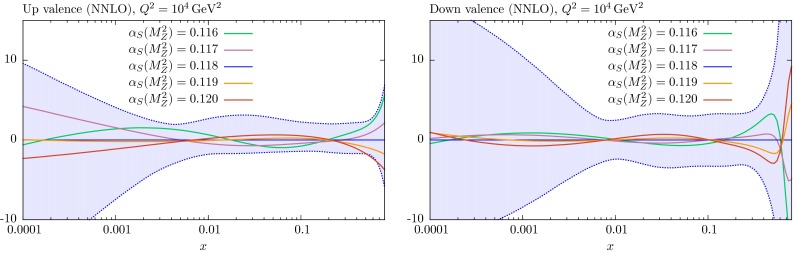
Percentage difference in the NNLO up and down valence quark PDFs at $Q^2=10^4$ $\mathrm{GeV}^2$ relative to central ($\alpha _S(M_Z^2)=0.118$) set for fits with different values of $\alpha _S$, with the percentage error bands for the central set also shown

It is informative to see the changes in the PDFs obtained in global fits for fixed values of $\alpha _S(M_Z^2)$ relative to those obtained for the central value; we only consider the NNLO case here, but note that the NLO PDFs behave in a similar way. These are shown in Figs. 13, 14 and 15 for the various PDFs as a function of *x* for $Q^2=10^4$ GeV$^2$ – a value of $Q^2$ relevant to data from the LHC. In almost every case the changes in the PDFs for the coupling varied in the range $0.116<\alpha _S(M_Z^2)<0.120$ are well within the PDF uncertainty bounds.

As expected, the gluon distribution for $x<0.1$ is larger for $\alpha _S(M_Z^2)=0.116$ and smaller for $\alpha _S(M_Z^2)=0.120$: a change which preserves the product $\alpha _S g$, which approximately determines the evolution of $F_2(x,Q^2)$ with $Q^2$ at low *x*. This is the dominant constraint on the gluon, and a smaller low *x* gluon leads to a larger high-*x* gluon (and *vice versa*) due to the momentum sum rule. The *u* and *d* PDFs have the opposite trend as $\alpha _S(M_Z^2)$ changes. At small *x* values this is a marginal effect, due to the interplay of a variety of competing elements. At high *x* the decreasing quark distribution with increasing $\alpha _S$ is due to the quicker evolution of quarks to lower *x*. The insensitivity of the strange-quark PDF to variations of $\alpha _S(M_Z^2)$ at low *x* is partly just due to the relative insensitivity of all low-*x* quarks, but is also partially explained by the comments in the previous section about the MMHT analysis [4] of dimuon production in neutrino interactions – where the changes in $\alpha _S(M_Z^2)$ are, to some extent, compensated by changes in the $B(D\rightarrow \mu )$ branching ratio parameter.

**Fig. 16:**
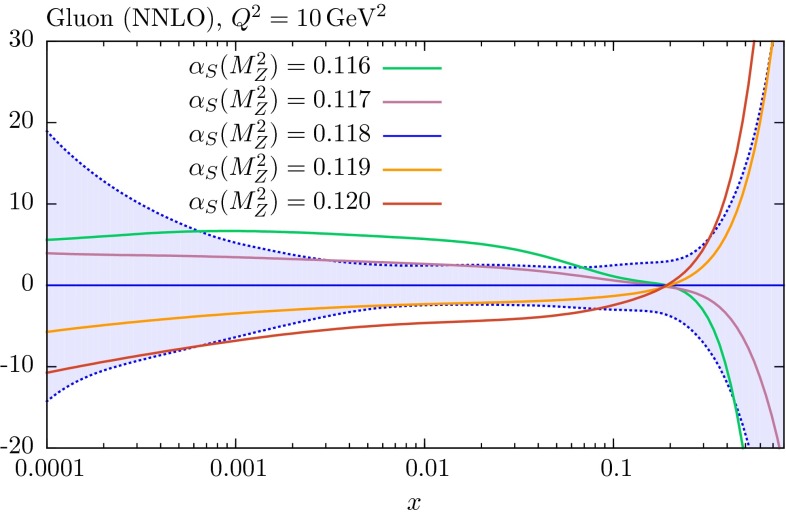
Percentage difference in the NNLO gluon PDFs at $Q^2=10$ $\mathrm{GeV}^2$ relative to central ($\alpha _S(M_Z^2)=0.118$) set for fits with different values of $\alpha _S$, with the percentage error bands for the central set also shown

In Fig. 16 we compare the changes in the gluon PDF for different fixed values of $\alpha _S(M_Z^2)$ at a much lower value of $Q^2$, namely $Q^2=10$ GeV$^2$. Here the gluon PDF is much more sensitive to the value of $\alpha _S(M_Z^2)$, and the changes in the gluon PDF lie outside its uncertainty bounds. The message is clear. At the high value of $Q^2=10^4$ GeV$^2$ the long evolution length means that the gluon PDF in the relevant broad *x* range about $x\sim 0.01$ is determined by PDFs at larger *x*, and is relatively insensitive to the parameters of the starting distributions.

## Benchmark cross sections

In this section we show uncertainties for cross sections at the Tevatron, and for 7 and $14~\mathrm TeV$ at the LHC. Uncertainties for 8 and $13~\mathrm TeV$ will be very similar to those at 7 and $14~\mathrm TeV$, respectively. We calculate the cross sections for *W* and *Z* boson, Higgs boson via gluon–gluon fusion and top-quark pair production.

We calculate the PDF and $\alpha _S(M_Z^2)$ uncertainties for the MMHT2014 PDFs [4] at the default values of $\alpha _S(M_Z^2)$. We use a value of $\Delta \alpha _S(M_Z^2)=0.001$ as an example, simply because PDF sets are readily available with $\alpha _S(M_Z^2)$ changes in units of 0.001. However, for values similar to $\Delta \alpha _S(M_Z^2)=0.001$ a linear scaling of the uncertainty can be applied to a very good approximation. As explained in Sect. 5, the full PDF+$\alpha _S(M_Z^2)$ uncertainty may then be obtained by adding the two uncertainties in quadrature.

To calculate the cross section we use the same procedure as was used in [4]. That is, for *W*, *Z* and Higgs production we use the code provided by Stirling, based on the calculation in [19–21], and for top pair production we use the procedure and code of [22]. Here our primary aim is not to present definitive predictions or to compare in detail to other PDF sets, as both these results are frequently provided in the literature with very specific choices of codes, scales and parameters which may differ from those used here. Rather, our main objective is to illustrate the procedure for estimating realistic PDF$+\alpha _S(M_Z^2)$ uncertainties.

### *W* and *Z* production

We begin with the predictions for the *W* and *Z* production cross sections. The results at NNLO are shown in Table 1. In this case the cross sections contain zeroth-order contributions in $\alpha _S$, with positive NLO corrections of about $20\,\%$, and much smaller NNLO contributions. Hence a smaller than $1\,\%$ change in $\alpha _S(M_Z^2)$ will only directly increase the cross section by a small fraction of a percent. The PDF uncertainties on the cross sections are $2\,\%$ at the Tevatron and slightly smaller at the LHC – the lower beam energy at the Tevatron meaning the cross sections have higher contribution from higher *x* where PDF uncertainties increase. The $\alpha _S$ uncertainty is small, about $0.6\,\%$ at the Tevatron and close to $1\,\%$ at the LHC, being slightly larger at 14 TeV than at 7 TeV. Hence, the $\alpha _S$ uncertainty is small, but more than the small fraction of a percent expected from the direct change in the cross section with $\alpha _S$. In fact the main increase in cross sections with $\alpha _S$ is due to the change in the PDFs with the coupling, rather than its direct effect on the cross section. From Fig. 14 we see that the up and down quark PDFs increase with $\alpha _S$ below $x\sim 0.1$–0.2 due to increased speed of evolution. From Fig. 13 we note that the strange-quark PDF increases a little with $\alpha _S$ at all *x* values. As already mentioned the Tevatron cross sections are more sensitive to the high-*x* quarks, which decrease with increasing $\alpha _S$, so this introduces a certain amount of anti-correlation of the cross section with $\alpha _S$. However, the main contribution is from a sufficiently low enough *x* that the distributions increase with $\alpha _S$, so the net effect is an increase with $\alpha _S$ a little larger than that coming directly from the $\alpha _s$ dependence of the cross section. As the energy increases at the LHC the contributing quarks move on average to lower *x* and the increase of the cross section with $\alpha _S$ increases – very slightly more so at 14 TeV than at 7 TeV. However, even at 14 TeV the total PDF+$\alpha _S$ uncertainty obtained by adding the two contributions in quadrature, is only a maximum of about $25\,\%$ greater (for $W^-$) than the PDF uncertainty alone if $\Delta \alpha _S(M_Z^2)= 0.001$ is used.

**Table 1:** Predictions for $W^\pm$ and *Z* cross sections (in nb), including leptonic branching, obtained with the NNLO MMHT2014 parton sets. The PDF and $\alpha _S$ uncertainties are also shown, where the $\alpha _S$ uncertainty corresponds to a variation of $\pm 0.001$ around its central value. The full PDF$+\alpha _S(M_Z^2)$ uncertainty is obtained by adding these two uncertainties in quadrature, as explained in Sect. 5

	$\sigma$	PDF unc.	$\alpha _S$ unc.
$W\,\, \mathrm{Tevatron}\,\,(1.96~\mathrm TeV)$	2.782	${}^{+0.056}_{-0.056}$ $\left( {}^{+2.0\,\%}_{-2.0\,\%}\right)$	${}^{+0.018}_{-0.020}$ $\left( {}^{+0.65\,\%}_{-0.72\,\%}\right)$
$Z \,\,\mathrm{Tevatron}\,\,(1.96~\mathrm TeV)$	0.2559	${}^{+0.0052}_{-0.0046}$ $\left( {}^{+2.0\,\%}_{-1.8\,\%}\right)$	${}^{+0.0015}_{-0.0018}$ $\left( {}^{+0.59\,\%}_{-0.70\,\%}\right)$
$W^+ \,\,\mathrm{LHC}\,\, (7~\mathrm TeV)$	6.197	${}^{+0.103}_{-0.092}$ $\left( {}^{+1.7\,\%}_{-1.5\,\%}\right)$	${}^{+0.058}_{-0.065}$ $\left( {}^{+0.94\,\%}_{-1.0\,\%}\right)$
$W^- \,\,\mathrm{LHC}\,\, (7~\mathrm TeV)$	4.306	${}^{+0.067}_{-0.076}$ $\left( {}^{+1.6\,\%}_{-1.8\,\%}\right)$	${}^{+0.043}_{-0.043}$ $\left( {}^{+1.0\,\%}_{-1.0\,\%}\right)$
$Z \,\,\mathrm{LHC}\,\, (7~\mathrm TeV)$	0.9638	${}^{+0.014}_{-0.013}$ $\left( {}^{+1.5\,\%}_{-1.3\,\%}\right)$	${}^{+0.0091}_{-0.010}$ $\left( {}^{+0.94\,\%}_{-1.0\,\%}\right)$
$W^+ \,\,\mathrm{LHC}\,\, (14~\mathrm TeV)$	12.48	${}^{+0.22}_{-0.18}$ $\left( {}^{+1.8\,\%}_{-1.4\,\%}\right)$	${}^{+0.12}_{-0.14}$ $\left( {}^{+0.97\,\%}_{-1.1\,\%}\right)$
$W^- \,\,\mathrm{LHC}\,\, (14~\mathrm TeV)$	9.32	${}^{+0.15}_{-0.14}$ $\left( {}^{+1.6\,\%}_{-1.5\,\%}\right)$	${}^{+0.098}_{-0.11}$ $\left( {}^{+1.1\,\%}_{-1.2\,\%}\right)$
$Z \,\,\mathrm{LHC}\,\, (14~\mathrm TeV)$	2.065	${}^{+0.035}_{-0.030}$ $\left( {}^{+1.7\,\%}_{-1.5\,\%}\right)$	${}^{+0.020}_{-0.025}$ $\left( {}^{+0.97\,\%}_{-1.2\,\%}\right)$

### Top-quark pair production

In Table 2 we show the analogous results for the top-quark pair production cross section. At the Tevatron the PDFs are probed in the region $x\approx 0.4/1.96\approx 0.2$, and the main production is from the $q{\bar{q}}$ channel. As we saw, the quark distributions are reasonably insensitive to $\alpha _S(M_Z^2)$ in this region of *x*, as it is the approximate pivot point of the PDFs. Hence, there is only a small change in cross section due to changes in the PDFs with $\alpha _S$. However, the cross section for $t{\bar{t}}$ production begins at order $\alpha _S^2$, and there is a significant positive higher-order correction at NLO and still an appreciable one at NNLO. Therefore, a change in $\alpha _S$ a little lower than $1\,\%$ should give a direct change in the cross section of about $2\,\%$. This is roughly the change that is observed. This is compared to a PDF-only uncertainty of nearly $3\,\%$ due to sensitivity to higher *x* quarks that occurs for *W*, *Z* production.

**Table 2:** Predictions for $t\overline{t}$ cross sections (in nb), obtained with the NNLO MMHT2014 parton sets. The PDF and $\alpha _S$ uncertainties are also shown, where the $\alpha _S$ uncertainty corresponds to a variation of $\pm 0.001$ around its central value. The full PDF$+\alpha _S(M_Z^2)$ uncertainty is obtained by adding these two uncertainties in quadrature, as explained in Sect. 5

	$\sigma$	PDF unc.	$\alpha _S$ unc.
$t\overline{t}$ $\mathrm{Tevatron}\,\,(1.96~\mathrm TeV)$	7.51	${}^{+0.21}_{-0.20}$ $\left( {}^{+2.8\,\%}_{-2.7\,\%}\right)$	${}^{+0.17}_{-0.15}$ $\left( {}^{+2.3\,\%}_{-2.1\,\%}\right)$
$t\overline{t}$ $\mathrm{LHC}\,\, (7~\mathrm TeV)$	175.9	${}^{+3.9}_{-5.5}$ $\left( {}^{+2.2\,\%}_{-3.1\,\%}\right)$	${}^{+4.1}_{-3.3}$ $\left( {}^{+2.3\,\%}_{-1.9\,\%}\right)$
$t\overline{t}$ $\mathrm{LHC}\,\, (14~\mathrm TeV)$	969.9	${}^{+16}_{-20}$ $\left( {}^{+1.6\,\%}_{-2.1\,\%}\right)$	${}^{+16}_{-14}$ $\left( {}^{+1.6\,\%}_{-1.4\,\%}\right)$

At the LHC the dominant production at higher energies (and with a proton–proton rather than proton–antiproton collider) is gluon–gluon fusion, with the central *x* value probed being $x\approx 0.4/7 \approx 0.06$ at 7 TeV, and $x\approx 0.4/14\approx 0.03$ at 14 TeV. As seen from the left plot of Fig. 13 the gluon decreases with increasing $\alpha _S(M_Z^2)$ below $x=0.1$ and the maximum decrease is for $x\sim 0.02-0.03$. The $\alpha _S(M_Z^2)$ uncertainty on $\sigma _{t \bar{t}}$ for 7 TeV is about $2\,\%$, almost as large as at the Tevatron, with the gluon above the pivot point still contributing considerably to the cross section, so the indirect $\alpha _S(M_Z^2)$ uncertainty due to PDF variation largely cancels. For 14 TeV the lower *x* probed means that most contribution is below the pivot point and there is some anti-correlation between the direct $\alpha _S$ variation and the indirect, with a reduced $\alpha _S$ uncertainty of $1.5\,\%$. At this highest energy the PDF-only uncertainty has also reduced to about $2\,\%$ due to the decreased sensitivity to the uncertainty in high-*x* PDFs, the gluon in this case. At the Tevatron and for 7 TeV at the LHC the $\alpha _S(M_Z^2)$ uncertainty is a little smaller than the PDF uncertainty, and the total is about 1.3 times the PDF uncertainty alone. At 14 TeV they are very similar in size, so the total uncertainty, for $\Delta \alpha _S(M_Z^2)= 0.001$ is about $\sqrt{2}$ that of the PDF uncertainty.

### Higgs boson production

In Table 3 we show the uncertainties in the rate of Higgs boson production from gluon–gluon fusion. Again, the cross section starts at order $\alpha ^2_S$ and there are large positive NLO and NNLO contributions. Hence, changes in $\alpha _S$ of about $1\,\%$ would be expected to lead to direct changes in the cross section of about $3\,\%$. However, even at the Tevatron the dominant *x* range probed, i.e. $x\approx 0.125/1.96 \approx 0.06$, corresponds to a region where the gluon distribution falls with increasing $\alpha _S(M_Z^2)$ and at the LHC where $x \approx 0.01$–0.02 at central rapidity the anti-correlation between $\alpha _S(M_Z^2)$ and the gluon distribution is near its maximum. Hence, at the Tevatron the total $\alpha _S(M_Z^2)$ uncertainty is a little less than the direct value at a little more than $2\,\%$, and at the LHC it is reduced to $1.5\,\%$. In the former case this is a little less than the PDF uncertainty of $\sim$3 %, with some sensitivity to the relatively poorly constrained high-*x* gluon, while at the LHC the PDF uncertainty is much reduced due to the smaller *x* probed, and is similar to the $\alpha _S(M_Z^2)$ uncertainty. Hence for $\Delta \alpha _S(M_Z^2)= 0.001$ the uncertainty on the Higgs boson cross section from gluon–gluon fusion is about $\sqrt{2}$ that of the PDF uncertainty alone.

**Table 3:** Predictions for $t\overline{t}$ cross sections (in nb), obtained with the NNLO MMHT2014 parton sets. The PDF and $\alpha _S$ uncertainties are also shown, where the $\alpha _S$ uncertainty corresponds to a variation of $\pm 0.001$ around its central value. The full PDF$+\alpha _S(M_Z^2)$ uncertainty is obtained by adding these two uncertainties in quadrature, as explained in Sect. 5

	$\sigma$	PDF unc.	$\alpha _S$ unc.
$t\overline{t}$ $\mathrm{Tevatron}\,\,(1.96~\mathrm TeV)$	7.51	${}^{+0.21}_{-0.20}$ $\left( {}^{+2.8\,\%}_{-2.7\,\%}\right)$	${}^{+0.17}_{-0.15}$ $\left( {}^{+2.3\,\%}_{-2.1\,\%}\right)$
$t\overline{t}$ $\mathrm{LHC}\,\, (7~\mathrm TeV)$	175.9	${}^{+3.9}_{-5.5}$ $\left( {}^{+2.2\,\%}_{-3.1\,\%}\right)$	${}^{+4.1}_{-3.3}$ $\left( {}^{+2.3\,\%}_{-1.9\,\%}\right)$
$t\overline{t}$ $\mathrm{LHC}\,\, (14~\mathrm TeV)$	969.9	${}^{+16}_{-20}$ $\left( {}^{+1.6\,\%}_{-2.1\,\%}\right)$	${}^{+16}_{-14}$ $\left( {}^{+1.6\,\%}_{-1.4\,\%}\right)$

We also repeat the study at NLO for the Higgs cross section. The results are shown in Tables 4 and 5 for the central values of $\alpha _S(M_Z^2)=0.120$ and $\alpha _S(M_Z^2)=0.118$, respectively. The uncertainties are very different in the two cases, with the central values of the cross sections being about $3\,\%$ lower for $\alpha _S(M_Z^2)=0.118$ than for $\alpha _S(M_Z^2)=0.120$. Both sets of predictions are about $30\,\%$ lower than at NNLO, highlighting the large NNLO correction for this process. The PDF uncertainties are very similar to those at NNLO, though a little larger in detail. However, the $\alpha _S(M_Z^2)$ uncertainties are noticeably reduced, as the large variation in the NNLO ($\mathcal{O}(\alpha _S^4)$) cross section with $\alpha _S$ is now absent.

**Table 4:** Predictions for the Higgs boson cross sections (in nb), obtained with the NNLO MMHT 2014 parton sets. The PDF and $\alpha _S$ uncertainties are also shown, where the $\alpha _S$ uncertainty corresponds to a variation of $\pm 0.001$ around its central value. The full PDF$+\alpha _S(M_Z^2)$ uncertainty is obtained by adding these two uncertainties in quadrature, as explained in Sect. 5

	$\sigma$	PDF unc.	$\alpha _S$ unc.
Higgs $\mathrm{Tevatron}\,\,(1.96~\mathrm TeV)$	0.874	${}^{+0.024}_{-0.030}$ $\left( {}^{+2.7\,\%}_{-3.4\,\%}\right)$	${}^{+0.022}_{-0.018}$ $\left( {}^{+2.5\,\%}_{-2.1\,\%}\right)$
Higgs $\mathrm{LHC}\,\, (7~\mathrm TeV)$	14.56	${}^{+0.21}_{-0.29}$ $\left( {}^{+1.4\,\%}_{-2.0\,\%}\right)$	${}^{+0.23}_{-0.22}$ $\left( {}^{+1.6\,\%}_{-1.5\,\%}\right)$
Higgs $\mathrm{LHC}\,\, (14~\mathrm TeV)$	47.69	${}^{+0.63}_{-0.88}$ $\left( {}^{+1.3\,\%}_{-1.8\,\%}\right)$	${}^{+0.71}_{-0.70}$ $\left( {}^{+1.5\,\%}_{-1.5\,\%}\right)$

**Table 5:** Predictions for Higgs Boson cross sections (in nb), obtained with the NLO MMHT 2014 parton sets. The PDF and $\alpha _s$ are shown, with the $\alpha _s$ uncertainty corresponding to a variation of $\pm 0.001$ around the central value ($\alpha _S(M_Z^2)=0.120$). The full PDF$+\alpha _S(M_Z^2)$ uncertainty is obtained by adding these two uncertainties in quadrature, as explained in Sect. 5

	$\sigma$	PDF unc.	$\alpha _S$ unc.
Higgs $\mathrm{Tevatron}\,\,(1.96~\mathrm TeV)$	0.644	${}^{+0.021}_{-0.022}$ $\left( {}^{+3.3\,\%}_{-3.4\,\%}\right)$	${}^{+0.011}_{-0.0088}$ $\left( {}^{+1.7\,\%}_{-1.4\,\%}\right)$
Higgs $\mathrm{LHC}\,\, (7~\mathrm TeV)$	11.28	${}^{+0.21}_{-0.20}$ $\left( {}^{+1.9\,\%}_{-1.8\,\%}\right)$	${}^{+0.15}_{-0.14}$ $\left( {}^{+1.3\,\%}_{-1.2\,\%}\right)$
Higgs $\mathrm{LHC}\,\, (14~\mathrm TeV)$	37.63	${}^{+0.67}_{-0.59}$ $\left( {}^{+1.8\,\%}_{-1.6\,\%}\right)$	${}^{+0.51}_{-0.50}$ $\left( {}^{+1.4\,\%}_{-1.3\,\%}\right)$

## Conclusions

The PDFs determined from global fits to deep-inelastic and related hard-scattering data are highly correlated to the value of $\alpha _S(M_Z^2)$ used, and any changes in the values of $\alpha _S(M_Z^2)$ must be accompanied by changes in the PDFs such that the optimum fit to data is still obtained. In [4] we produced PDF and uncertainty eigenvector sets for specific values of $\alpha _S(M_Z^2)$, guided by the values obtained when it was left as a free parameter in the fit. In this article we explicitly present PDF sets and the global fit quality at NLO and NNLO for a wide variety of $\alpha _S(M_Z^2)$ values, i.e. $\alpha _S(M_Z^2)=0.108$ to $\alpha _S(M_Z^2)=0.128$ in steps of $\Delta \alpha _S(M_Z^2)=0.001$. Hence, we illustrate in more detail the origin of our best fit $\alpha _S(M_Z^2)$ values of

7 $$\begin{aligned}&\text {NLO:}\qquad \alpha _S(M_Z^2) = 0.1201 \pm 0.0015 \text { (68\,\% C.L.)},\end{aligned}$$

8 $$\begin{aligned}&\text {NNLO:}\qquad \alpha _S(M_Z^2) = 0.1172 \pm 0.0013 \text { (68\,\% C.L.)}, \end{aligned}$$

already presented in [4], but also present the uncertainties. We show the variation of the fit quality with $\alpha _S(M_Z^2)$ of each data set, within the context of the global fit, and see which are the more and less constraining sets, and which prefer higher and lower values. We see that most data sets show a systematic trend of preferring a slightly lower $\alpha _S(M_Z^2)$ value at NNLO than at NLO, but note that no particular type of data strongly prefers a high or low value of $\alpha _S(M_Z^2)$. HERA and Tevatron data tend to prefer higher values, but are not the most constraining data. There are examples of fixed target DIS data which prefer either high or low values and similarly for the LHC data sets, which are new compared to our previous analysis [13]. Indeed our best values of $\alpha _S(M_Z^2)$ are almost unchanged from $\alpha _S(M_Z^2)=0.1202$ (NLO) and $\alpha _S(M_Z^2)=0.1171$ (NNLO). They are also very similar to the values obtained by NNPDF of $\alpha _S(M_Z^2)=0.1191$ (NLO)[23] and $\alpha _S(M_Z^2)=0.1173$ (NNLO) [24]. However, our extraction disagrees with the recent value $\alpha _S(M_Z^2)=0.1132$ (NNLO) in [25]. We find agreement at the level of one sigma or less with the world average value of $\alpha _S(M_Z^2)=0.1187 \pm 0.0005$, and this improves when we include the world average (without the DIS determinations included) as a data point in our fit, when we obtain $\alpha _S(M_Z^2)=0.1195$ (NLO) and $\alpha _S(M_Z^2)=0.1178$ (NNLO). Hence, our NNLO value including $\alpha _S(M_Z^2)$ as an external constraint is in excellent agreement with the preferred value, $\alpha _S(M_Z^2)=0.118$, for which eigenvector sets are made available. The PDF sets obtained at the 21 different values of $\alpha _S(M_Z^2)$ at NLO and NNLO can be found at [26] and are available from the LHAPDF library [27]. They should be useful in studies of $\alpha _S(M_Z^2)$ by other groups.

In order to calculate the PDF$+\alpha _S(M_Z^2)$ uncertainty we now advocate the approach pioneered in [18] of treating PDFs with $\alpha _S(M_Z^2)\pm \Delta \alpha _S(M_Z^2)$ as an extra eigenvector set. As shown in [18], provided certain conditions are met (at least approximately), the $\alpha _S(M_Z^2)$ uncertainty may be correctly added to the PDF uncertainty by simply adding in quadrature the variation of any quantity under a change in coupling $\Delta \alpha _S(M_Z^2)$ as long as the change in $\alpha _S(M_Z^2)$ is accompanied by the appropriate change in PDFs required by the global fit. As examples, we have calculated the total cross sections for the production of *W*, *Z*, top-quark pairs and Higgs bosons at the Tevatron and LHC. For *W* and *Z* production, where the LO subprocess is $\mathcal{O}(\alpha _S^0)$ and is quark-initiated, the combined “PDF+$\alpha _S$” uncertainty is not much larger than the PDF-only uncertainty with a fixed $\alpha _S$. However, the additional uncertainty due to $\alpha _S$ is more important for top-quark pair production and Higgs boson production via gluon–gluon fusion, since the LO subprocess now is $\mathcal{O}(\alpha _S^2)$, though the details depend on the correlation between $\alpha _S(M_Z^2)$ and the contributing PDFs.

In addition, we note that for any particular process the details of the uncertainty can now be explicitly calculated in a straightforward way using the PDFs we have provided in this paper, together with the procedure for combining PDF and $\alpha _S(M_Z^2)$ uncertainty discussed in Sect. 5.

Moreover, it is also straightforward to apply the procedure to determine the uncertainties coming from combinations of PDF sets obtained by global analyses of different groups. Using techniques given in [28–31] it is possible to combine different PDF sets at a preferred value of $\alpha _S(M_Z^2)$ such that the central value and the uncertainty of the combination are correctly obtained. The procedure to determine the uncertainty due to variations of $\alpha _S(M_Z^2)$ is as follows. If each group used in the combination also makes available sets of PDFs obtained by repeating their global fits^5^ with $\alpha _S(M_Z^2)\pm \Delta \alpha _S(M_Z^2)$, then an additional pair of PDF sets representing the $\alpha _S(M_Z^2)$ variation of the combination can be obtained just by taking the average of the PDFs from each group obtained at $\alpha _S(M_Z^2)+ \Delta \alpha _S(M_Z^2)$, and by taking the average at $\alpha _S(M_Z^2)- \Delta \alpha _S(M_Z^2)$. As a result the PDF$+\alpha _S(M_Z^2)$ uncertainty for any quantity calculated using the combined set is just the PDF induced uncertainty at the preferred value of $\alpha _S(M_Z^2)$ added in quadrature to the $\alpha _S(M_Z^2)$ uncertainty determined from the two combined sets defined at $\alpha _S(M_Z^2)\pm \Delta \alpha _S(M_Z^2)$. Hence, a user may determine for any process the optimum prediction, the PDF uncertainty, the $\alpha _S(M_Z^2)$ uncertainty and the complete PDF$+\alpha _S(M_Z^2)$ uncertainty arising from the combination of a whole collection of different PDFs.

## Footnotes

1 In the $\overline{\mathrm{MS}}$ scheme this involves discontinuities at flavour transition points at NNLO. For a suggestion for a smooth transition in a physical scheme see [12].

2 Our constraint on $\alpha _S(M_Z^2)$ is very consistent with that in [17].

3 The constraint from $\sigma _{t \bar{t}}$ data does provide the dominant constraint in one direction for eigenvector 15 at NNLO. However, very nearly as strong a constraint is provided by other data sets and the eigenvector only provides at the very most 40 % of the uncertainty on one distribution, the gluon, at any *x* value, in practice at high *x*. Hence, a slightly increased tolerance for this eigenvector would have a minimal impact on any PDF uncertainties.

4 Taking a weighted average of the values in Eqs. (4) and (5) would result in values slightly nearer to the world average, reflecting the fact that the dynamical tolerance procedure used to determine the uncertainty results in a $\Delta \chi ^2_\mathrm{global} > 1$.

5 For instance, if $\alpha _S(M_Z^2)=0.118$ is the preferred value then repeating global fits at $\alpha _S(M_Z^2)=0.117$ and $\alpha _S(M_Z^2)=0.119$ would be sufficient to quantify the uncertainty due variations of $\alpha _S$.
